# Putative Transcriptomic Biomarkers in the Inflammatory Cytokine Pathway Differentiate Major Depressive Disorder Patients from Control Subjects and Bipolar Disorder Patients

**DOI:** 10.1371/journal.pone.0091076

**Published:** 2014-03-11

**Authors:** Timothy R. Powell, Peter McGuffin, Ursula M. D'Souza, Sarah Cohen-Woods, Georgina M. Hosang, Charlotte Martin, Keith Matthews, Richard K. Day, Anne E. Farmer, Katherine E. Tansey, Leonard C. Schalkwyk

**Affiliations:** 1 King's College London, Institute of Psychiatry, MRC Social, Genetic and Developmental Psychiatry Centre, London, United Kingdom; 2 Discipline of Psychiatry, University of Adelaide, Adelaide, Australia; 3 Division of Neuroscience, Ninewells Hospital and Medical School, Dundee, United Kingdom; Chiba University Center for Forensic Mental Health, Japan

## Abstract

Mood disorders consist of two etiologically related, but distinctly treated illnesses, major depressive disorder (MDD) and bipolar disorder (BPD). These disorders share similarities in their clinical presentation, and thus show high rates of misdiagnosis. Recent research has revealed significant transcriptional differences within the inflammatory cytokine pathway between MDD patients and controls, and between BPD patients and controls, suggesting this pathway may possess important biomarker properties. This exploratory study attempts to identify disorder-specific transcriptional biomarkers within the inflammatory cytokine pathway, which can distinguish between control subjects, MDD patients and BPD patients. This is achieved using RNA extracted from subject blood and applying synthesized complementary DNA to quantitative PCR arrays containing primers for 87 inflammation-related genes. Initially, we use ANOVA to test for transcriptional differences in a ‘discovery cohort’ (total n = 90) and then we use t-tests to assess the reliability of any identified transcriptional differences in a ‘validation cohort’ (total n = 35). The two most robust and reliable biomarkers identified across both the discovery and validation cohort were Chemokine (C-C motif) ligand 24 (*CCL24*) which was consistently transcribed higher amongst MDD patients relative to controls and BPD patients, and C-C chemokine receptor type 6 (*CCR6*) which was consistently more lowly transcribed amongst MDD patients relative to controls. Results detailed here provide preliminary evidence that transcriptional measures within inflammation-related genes might be useful in aiding clinical diagnostic decision-making processes. Future research should aim to replicate findings detailed in this exploratory study in a larger medication-free sample and examine whether identified biomarkers could be used prospectively to aid clinical diagnosis.

## Introduction

The term ‘mood disorder’ refers to a category of psychiatric illness that is characterized by a pathological distortion of affect [1]. Mood disorders represent the most common form of severe adult-onset psychiatric disorder and are predicted to be the second most common cause of morbidity by 2020 [2], [3]. They consist of two etiologically related [4] but distinctly treated psychiatric illnesses [5], major depressive disorder (MDD) and bipolar disorder (BPD). Both MDD and BPD are clinically characterized by episodes of depression (e.g. lowered mood, loss of interest or pleasure, loss of energy); with BPD also consisting of episodes of mania or hypomania (e.g. expanded self-esteem, increased distractibility, talkativeness) [6], [7].

Despite the establishment of clinical diagnostic criteria for MDD and BPD, the heterogeneous nature of these disorders, the similarities they share in their clinical presentation, and the absence of specific biomarkers, means there are relatively high rates of misdiagnosis [8], [9]. BPD is often misdiagnosed in the first instance [10], and an estimated 5.7 years on average is required for the correct diagnosis [11]. Most frequently, BPD is misdiagnosed as MDD due to their overlapping symptomology, the often later onset of mania, and more frequent occurrence of depressive episodes in BPD patients [12], [13]. Misdiagnosis may be particularly high when BPD patients present symptoms indicative of a clinically significant depressive episode but are premorbid for manic symptoms, or have failed to recognize previous manic states. Misdiagnosis, and therefore incorrect treatment of BPD with monotherapy antidepressant treatment, increases the risk of antidepressant induced mania [14], [15] and “cycle acceleration” (an increased frequency of episodes) [13]; both of which can have damaging effects on disease prognosis. Consequently, the establishment of biomarkers specific to each disorder remains a key goal, so that the correct diagnosis and treatment can be obtained for a patient from the outset.

The clear need for an objective, empirical method of diagnosis has led to genome-wide association studies (GWASs) attempting to identify genes associated with MDD and BPD. However, despite twin studies suggesting mood disorders are moderately heritable, GWASs have largely been unsuccessful in identifying genes robustly associated with MDD [4], [16], with only recent reports from very large-scale studies finding genes potentially being associated with BPD [17]. In addition to genetic background, it has been established that environmental factors, such as stressful life events, can also increase a person's susceptibility to developing a mood disorder, and precipitate mood disorder episodes [18], [19]. Subsequently, it has been proposed that a lack of findings from GWASs might relate to the more salient presence of gene-environment interactions [20], as supported by studies in the field [21]–[24]. Therefore, it may be the interface between genes and environment that contains the most valuable biomarker information about mood disorders, as opposed to genotype alone. Thus, focusing efforts on identifying biomarkers at the level of the transcriptome, which represents a functional molecular output of gene-environment interactions, might yield more fruitful results.

Cytokines are a group of cell-signaling proteins which, in the periphery, aid inflammatory processes and the immune system to form coordinated responses to infection [25]. Cytokines are also expressed centrally and have effects on the brain, influencing neurotransmitter systems, neuroendocrine function and neural plasticity, and converging evidence suggests they may play an important role in the pathophysiology of mood disorders [26]. Furthermore, cytokines can cross the blood-brain barrier [27], so peripheral cytokines may represent a potentially useful biomarker resource relating to mood disorders. Indeed, both protein and transcriptomic studies performed in blood have revealed differences in the expression of cytokines such as interleukin-6, tumor necrosis factor and interleukin-1β amongst MDD patients relative to controls [25], [28]–[32]. Similarly, the transcription of cytokines has been found to differentiate between BPD patients and controls [33]. However, no studies have yet investigated whether disorder-specific transcriptional differences exist within the inflammatory cytokine pathway, which might be used as clinical diagnostic aids to differentiate between MDD and BPD patients.

The current study aims to identify transcriptomic biomarkers in the inflammatory cytokine pathway which could be used to distinguish between controls subjects, MDD patients and BPD patients. We achieve this using RNA extracted from whole blood and examine an extensive set of inflammatory-related transcripts including genes coding for: interleukins and interleukin receptors, chemokines and chemokine receptors, the tumor necrosis factor cytokine family and receptors, and other inflammatory regulators. We initially test for differences in a discovery cohort (total n = 90), and then attempt to replicate any findings from our discovery cohort in a validation cohort (total n = 35).

## Methods

### Clinical samples

Patient samples used in this study were collected from two methodologically similar studies, the Bipolar Association Case–Control Study (BACCS) [34] and the Genome-based Therapeutic Drugs for Depression Project (GENDEP) [35].

#### (i) Bipolar Disorder Patients

BPD patients in BACCS were recruited from three sites, Toronto Canada, London UK and Dundee UK. BACCS was a community-based study, where subjects were recruited from psychiatric clinics, hospitals, primary care physicians and patient support groups. BPD patients were included in the study if they were over the age of 18 and had been diagnosed with Bipolar I or Bipolar II disorder as defined by the DSM-IV or ICD-10 [6], [7]. All patients were interviewed using the Schedules for Clinical Assessment in Neuropsychiatry (SCAN) [36]. All patients recruited in BACCS were euthymic (not in a clinically significant mood episode) at the time of interview and blood collection. All subjects were of White European parentage. Exclusion criteria include: first-degree relative having fulfilled criteria for schizophrenia; psychotic symptoms that were mood incongruent or present when there was no evidence for mood disturbance; intravenous drug use with a lifetime diagnosis of drug dependency; mania or depression occurring solely in relation to, or a consequence of, alcohol or substance abuse/dependence and/or medical illness; being related to an individual already included in the study.

The current study utilized 40 BPD patient samples in total (30 in the discovery cohort and 10 in the validation cohort) collected only from the Dundee UK site, as this was the only site to collect blood for transcriptomic experiments. The subset used here was randomly selected from a larger group of samples. Further patient characteristics are detailed in *Tables 1* and *2*, note that information on comorbidities and current medication use is based on self-reports at the time of blood collection.

**Table 1:** A summary of subject characteristics in our discovery cohort.

Subject Characteristic	BPD	MDD	Control	Total Sample
Sample number	30	30	30	90
Age (mean, (SD))*	53.10 (14.17)	41.23 (12.53)	52.40 (14.35)	48.91 (14.62)
Males (n)	10	10	9	29
Females (n)	20	20	21	61
BMI (mean, (SD))	26.66 (5.51)	25.90 (4.11)	24.93 (3.33)	25.83
Cardiovascular Problem (n)*	8	1	5	14
Diabetes (n)	2	0	2	4
Antidepressants (n)	6	30	0	36
Lithium (n)	20	0	0	20
Carbamazepine (n)	3	0	0	3
Sodium valproate (n)	3	0	0	3
Antipsychotics (n)	16	0	0	16

This includes general characteristics (total number in each subject group, age, number of males, number of females), information about co-morbidity (body mass index (BMI), number with diabetes, number with cardiovascular problems), and current medication use (antidepressants, antipsychotics, lithium, carbamazepine, and sodium valproate). Note: cardiovascular problems is an umbrella term consisting of those subjects who reported high levels of cholesterol, high blood pressure, or a history of angina or heart attacks. For age, males (n), females (n), BMI, cardiovascular problems (n), and diabetes (n) we performed ANOVA to assess differences between groups. Significant differences between groups (p≤0.05) is indicated with a *. [Age: F(2, 87) = 7.077, p = 0.001; Cardiovascular Problems: F(2, 87) = 3.252, p = 0.043].

**Table 2:** A summary of subject characteristics in our validation cohort.

Subject Characteristic	BPD	MDD	Control	Total Sample
Sample number	10	15	10	35
Age (mean, (SD))	52.50 (13.10)	45.19 (12.14)	54.6 (12.33)	49.83 (12.84)
Males (n)	2	7	3	12
Females (n)	8	8	7	23
BMI (mean, (SD))	24.68 (3.92)	26.01 (2.75)	28.33 (4.46)	26.22 (12.84)
Cardiovascular Problem (n)	3	4	0	7
Diabetes (n)	2	0	0	2
Antidepressants (n)	1	0	0	1
Lithium (n)	7	0	0	7
Carbamazepine (n)	2	0	0	2
Sodium valproate (n)	1	0	0	1
Antipsychotics (n)	3	0	0	3

This includes general characteristics (total number in each subject group, age, number of males, number of females), information about co-morbidity (body mass index (BMI), number with diabetes, number with cardiovascular problems), and current medication use (antidepressants, antipsychotics, lithium, carbamazepine, and sodium valproate). Note: cardiovascular problems is an umbrella term consisting of those subjects who reported high levels of cholesterol, high blood pressure, or a history of angina or heart attacks. For age, males (n), females (n), BMI, cardiovascular problems (n), and diabetes (n) we performed ANOVA to assess differences between groups. Significant differences between groups (p≤0.05) is indicated with a *.

#### (ii) Major Depressive Disorder Patients

MDD patient samples were collected as part of the European study GENDEP, which is a 12-week partially randomized open label pharmacogenetic study. Patients were selected if they were diagnosed with MDD of at least moderate severity according to ICD-10 or DSM-IV criteria [6], [7]. Patients in GENDEP were aged between 19–72 years and of White European parentage. Diagnoses were established using the semistructured SCAN interview [36]. Exclusion criteria included personal and family history of schizophrenia or bipolar disorder; current substance dependence; being related to an individual already included in the study; known treatment resistance to both of the antidepressants given as part of the study.

The current study utilizes 45 patient samples in total (30 in the discovery cohort and 15 in the validation cohort), which were randomly selected from the larger GENDEP sample set. Blood samples were collected both at the start of GENDEP and after eight weeks of treatment with escitalopram as described previously [37]. All patients completed the Beck Depression Inventory at the time of blood collection (BDI) [38]. We utilized blood collected after eight weeks of treatment with escitalopram, for both our discovery and validation cohorts. We chose this time point as it allowed us to adjust for the possible dynamic effects of current mood state on gene transcription, and so allowing us to accurately compare our MDD sample with our euthymic BPD patients and control subject sample. Unlike at the start of the GENDEP trial where all patient were in a clinically significant depressed state, after eight weeks of treatment, 26 patients still showed mild to moderate depression (defined here by BDI>10), whereas 19 patients were no longer in a clinically significant depressed state (defined here by BDI≤10). Furthermore, our previous work has revealed that escitalopram has no significant effect on the transcription of genes in the inflammatory cytokine pathway with the exception of ATP-binding cassette sub-family F member 1 (*ABCF1*) [39], which has been excluded as a potential biomarker. Subsequently, medication is unlikely to act as a confounding factor in this MDD sample. Further patient characteristics are shown in *Tables 1* and *2*, note that information on comorbidities and current medication use is based on self-reports at the time of blood collection.

We also utilized blood which was collected at the start of GENDEP to ascertain how stable transcriptional biomarkers were at differentiating MDD patients from other subject groups. At the start of GENDEP all patients had been drug-free for two weeks and were all in a clinically significant mood state (BDI>10). We assess whether transcripts identified in the discovery cohort and replicated in the validation cohort, continue to differentiate MDD patients from other subject groups when blood is collected at a different time point, during a different mood state, and during the absence of medication.

#### (iii) Control Subjects

Control subjects were selected from BACCS where they were screened for lifetime absence of psychiatric disorders using a modified version of the Past History Schedule [40]. All controls subjects were of White European parentage. Exclusion criteria were if they; or a first-degree relative, ever fulfilled criteria for BPD, MDD or any other psychiatric disorder; if they had a BDI score of greater than 10 [38]; did not return consent; failed to return cheek swabs or successfully give blood. The current study utilized 40 subject blood samples in total (30 in discovery cohort and 10 in the validation cohort), collected only from the Dundee UK site, as this was the only site to collect blood for transcriptomic experiments. Further subject characteristics are shown in *Tables 1* *and* *2*, note that information on comorbidities and current medication use is based on self-reports at the time of blood collection.

### Ethics statement

The BACC and GENDEP studies were approved by The Joint South London and Maudsley NHS Trust Institute of Psychiatry Research Ethics Committee and at each participating centre and all subjects provided written informed consent.

### Experimental details

All blood samples from BACCS and GENDEP were collected in 10 ml PAXgene tubes (PreAnalytiX, Switzerland) and stored at −80°C. Prior to the start of gene expression studies, PAXgene tubes were allowed to thaw for 12 hours at room temperature. RNA extraction was performed using the Qiagen PAXgene Blood miRNA Kit (PreAnalytiX) following the standard manufacturer's protocol. The purity and quantity of RNA was measured using the Nanodrop, ND1000 (Thermoscientific, Wilmington, DE). All samples had 260/280 ratios of between 1.9 and 2.3. RNA integrity numbers (RINs) were furthermore assessed using the Agilent 2100 Bioanalyzer (Agilent Technologies, Berkshire, UK) and the average RIN was 8±1.5.

Reagents used in the quantitative PCR (qPCR) component of the study were manufactured by SABiosciences (Frederick, MD, USA). Complementary DNA (cDNA) was prepared using 1 µg of total RNA and the SABiosciences RT^2^ HT First Strand Kit following the manufacturer's protocol. Briefly, following genomic DNA removal, the samples were incubated for 15 minutes at 42°C with 6 µl of BC4 RT Mastermix (SABiosciences). The reverse transcriptase enzyme was subsequently inactivated at 95°C for 5 minutes. cDNA samples generated were stored at −20°C prior to use in the qPCR experiments.

Customized 384-well arrays were designed for qPCR experiments. These arrays contained lyophilized primers for the 84 genes listed in the commercially available Human Inflammatory Cytokines & Receptors PCR Array (SABiosciences), with the addition of gene primers for interleukin 11 (*IL11*), interleukin-6 (*IL6*) and the glucocorticoid receptor (*NR3C1*). Each array contained five housekeeping genes for normalization. These include: β2-microglobulin (*B2M*), hypoxanthine phosphoribosyltransferase (*HPRT1*), ribosomal protein L13a (*RPL13A*), glyceraldehyde-3-phosphate dehydrogenase (*GAPDH*) and β-actin (*ACTB*). The three most stable housekeeping genes were selected based on RefFinder analyses and used for normalization across samples.

Each 384-well array was designed to analyze four samples simultaneously. The qPCR reagents used consisted of: 550 µl of 2X SABiosciences RT^2^ qPCR Master Mix (SYBR green), 102 µl of diluted synthesized cDNA and 448 µl RNAse free water, with a total volume of 1100 µl for each sample. Each qPCR array contained the following controls: human genomic DNA control (gDNA), reverse transcription control (RTC) and a positive PCR control (PPC). To ascertain whether samples passed quality control checks for gDNA and RTC, the manufacturer's quality control criteria were applied. The qPCR reactions were performed using the ABI Prism 7900HT Sequence Detection System (Applied Biosystems, California, USA). Thermal cycling conditions consisted of an enzyme activation stage (95°C for 10 minutes), followed by 40 cycles of a denaturation stage (95°C for 15 secs) and a hybridization and extension stage (65°C for 1 minute). The software program SDS 2.3 (Applied Biosystems) generated cycle threshold values (C*_t_*) from the data collected, see *Table S1* for raw C*_t_* values.

### Statistical Analysis

C*_t_* values of greater than 37 were removed and excluded from further analysis as such high C*_t_* values are indicative of very low expression levels. Furthermore, if as a result of data removal, a transcript showed missing data for more than half of the total patient sample, that transcript was excluded from further analysis. The relative expression of target genes was calculated by subtracting the mean C*_t_* of the selected reference genes from the C*_t_* of the target gene to generate ΔC*_t_* values [41]. As mood disorder pharmacotherapies can affect housekeeping gene expression, the three most stable housekeeping genes were selected as reference genes for normalization purposes based on RefFinder analyses (http://www.leonxie.com/referencegene.php) [42]. Relative expression values were then adjusted for PPC (to account for any inter-plate variability), age, sex, current mood status and the presence of comorbid disorders (diabetes, cardiovascular problems). Adjusted ΔC*_t_* were used in statistical calculations and adjusted 2^−ΔC*t*^ were used to generate plots [41].

To ascertain whether significant transcriptional differences existed between control, MDD and BPD subject groups in our discovery cohort, we performed analysis of variance (ANOVA) tests. Partial eta squared (η_p_^2^) was calculated as an estimate of effect size, by dividing the sum of squares between groups by the total sum of squares. Games-Howell post-hoc tests were subsequently performed to correct for multiple testing and to generate pair-wise comparisons between subject groups [43]. When making pairwise comparisons, Cohen's *d* was generated as our estimate of effect size, by calculating the mean differences between our two subject groups, and dividing this result by the square root of the within-groups mean square. Small (*d*≈0.2), medium (*d*≈0.5) and large effect sizes (*d*≥0.8) were then assumed, as according to Cohen [44]. Based on results from the discovery cohort, we then performed one-tailed independent sample t-tests in an attempt to replicate findings in our validation cohort. Similarly, Cohen's *d* was generated as our estimate of effect size by multiplying the t-test statistic value by two and dividing the result by the square root of the degrees of freedom [44].

We have previously shown that escitalopram does not affect the transcription of inflammatory cytokines in our MDD patient sample, with the exception of *ABCF1*, which has been excluded as a potential biomarker [39]. However, medications used in our BPD patient sample may affect transcription, and as such we performed post-hoc analyses to assess whether these medications may represent confounding factors. Consequently, for each gene's expression that significantly differentiated our BPD subjects from either controls or MDD patients, we ran univariate linear regressions for our BPD sample only. The expression of the significant gene was selected as the dependent variable and regularly used medications included as covariates. For any medications which significantly predicted the expression of a gene (p≤0.05), we excluded that transcript as a likely biomarker.

For any transcripts that significantly differentiated MDD patients from control subjects or BPD patients, in both the discovery and validation cohorts, we performed an additional test to determine the stability of these transcripts as state biomarkers for MDD. We achieved this by utilizing transcript data generated from blood collected at a different time point (start of GENDEP), under different conditions (patients were drug free, all patients were in a depressed episode). As before, we attempted to validate biomarkers by performing one-tail independent samples t-tests.

All statistical analyses were performed using SPSS Version 15 (SPSS Inc., Chicago, Illinois, USA). Graphs were generated using the ‘plot’ function in R (http://www.R-project.org).

## Results

### Validation of internal controls

All qPCR plates passed quality control checks outlined. 71 out of 87 target genes were sufficiently detectable according to out set criteria. RefFinder analyses revealed that *B2M*, *RPL13A* and *ACTB* were the three most stable housekeeping genes across all samples and subsequently were selected for normalization purposes, see *Figure S1*. Adjusted relative expression for all subjects can be found in *Table S2*.

### Transcriptional differences between subject groups

#### (i) Discovery Cohort

ANOVA revealed 11 genes which showed nominally significant transcriptional differences (p≤0.05) between our three subject groups. The most significant differences between subject groups were found in Chemokine (C-C motif) ligand 24 [*CCL24*: F (2, 85) = 6.438, p = 0.002, η_p_^2^ = 0.134] and interleukin-8 [*IL8*: F (2, 87) = 6.872, p = 0.002, η_p_^2^ = 0.136], see *Table S3* for full ANOVA results. Games-Howell post hoc analyses were subsequently performed on all genes present on the array. These tests correct for the effects of multiple testing, and generate pairwise comparisons, see *Table S4* for full results. Table 3 lists the genes which produced significant p-values from the ANOVA analyses (p≤0.05) and details corrected pair-wise results generated from Games-Howell post hoc analyses. None of the medications used by our BPD patients significantly affected the transcription of any of the potential biomarkers identified in our discovery cohort.

**Table 3:** A table detailing corrected Games-Howell pair-wise post-hoc analysis results for genes which produced significant p-values (p≤0.05) in ANOVA from our discovery cohort.

	Discovery Cohort																	
	MDD v Control						MDD v BPD						BPD v Controls					
Gene	Mean Difference	S.E.	p-value	95% C.I.		*d*	Mean Difference	S.E.	p-value	95% C.I.		*d*	Mean Difference	S.E.	p-value	95% C.I.		*d*
CCL24	−0.779	0.259	**0.011**	−1.404	−0.153	0.867	−0.676	0.249	**0.025**	−1.280	−0.072	0.753	−0.102	0.181	0.839	−0.538	0.334	0.114
CCR4	0.615	0.255	**0.049**	0.001	1.229	0.619	0.507	0.278	0.170	−0.161	1.175	0.511	0.108	0.236	0.891	−0.460	0.676	0.109
CCR6	0.510	0.192	**0.028**	0.047	0.973	0.815	0.091	0.215	0.907	−0.426	0.607	0.115	0.419	0.205	0.111	−0.074	0.913	0.530
CCR9	0.644	0.249	**0.032**	0.046	1.242	0.665	0.276	0.247	0.507	−0.318	0.870	0.285	0.368	0.256	0.327	−0.247	0.983	0.380
CXCL1	0.738	0.261	**0.017**	0.111	1.366	0.705	0.353	0.276	0.413	−0.311	1.017	0.337	0.385	0.274	0.345	−0.274	1.045	0.368
CXCL6	1.007	0.347	**0.015**	0.167	1.848	0.763	0.549	0.377	0.319	−0.358	1.456	0.416	0.459	0.294	0.270	−0.248	1.166	0.347
CXCL9	0.517	0.347	0.303	−0.318	1.352	0.428	1.004	0.317	**0.007**	0.239	1.770	0.831	−0.487	0.274	0.187	−1.149	0.174	0.403
CXCL10	1.094	0.372	**0.013**	0.197	1.991	0.718	0.944	0.421	0.072	−0.068	1.956	0.620	0.150	0.388	0.921	−0.784	1.084	0.100
XCR1	−0.109	0.226	0.881	−0.656	0.438	0.119	−0.772	0.262	**0.013**	−1.404	−0.141	0.849	0.664	0.214	**0.009**	0.148	1.180	0.729
IL8	1.021	0.303	**0.004**	0.292	1.749	0.839	0.026	0.323	0.997	−0.750	0.802	0.021	0.995	0.316	**0.007**	0.234	1.756	0.818
NR3C1	0.543	0.202	**0.025**	0.057	1.028	0.664	0.170	0.208	0.696	−0.332	0.672	0.208	0.373	0.222	0.221	−0.161	0.906	0.456

The table details results from pairwise comparisons between subject groups, including the mean differences in relative expression between subject groups, the standard error (S.E.), p-value, 95% confidence interval (95% C.I.), and Cohen's *d*. Significant pairwise comparisons (p≤0.05) are highlighted in bold.

#### (ii) Validation

**Figure 1:**
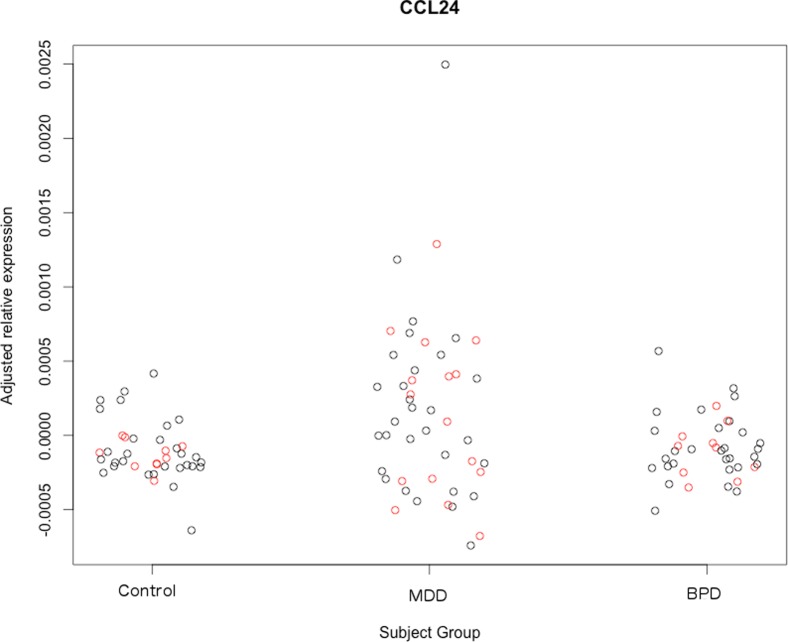
A plot showing the adjusted relative expression of *CCL24* (y-axis) in our control subjects, MDD subjects and BPD subjects (x-axis) using data collected from our discovery cohort (shown in black), and our validation cohort (shown in red). Note the higher transcription of *CCL24* in the MDD subject group relative to the control and BPD subject groups.

**Figure 2:**
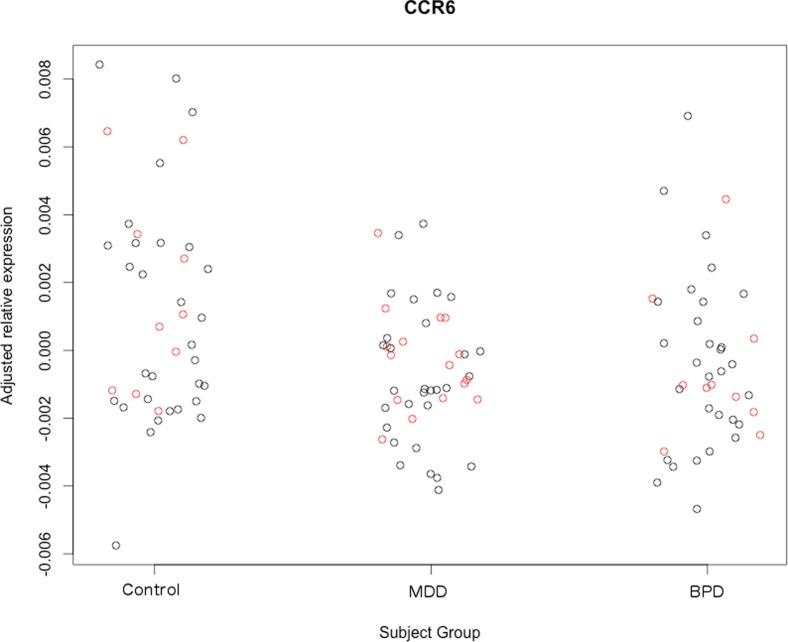
A plot showing the adjusted relative expression of *CCR6* (y-axis) in our control subjects, MDD subjects and BPD subjects (x-axis) using data collected from our discovery cohort (shown in black), and our validation cohort (shown in red). Note the lower transcription of *CCR6* in the MDD subject group relative to the control subject group.

One-tailed independent t-tests were used in our validation cohort to test whether we could replicate potential biomarkers identified from our discovery cohort, see Table 4. Higher transcription of *CCL24*, the most significant difference revealed between subjects in our discovery cohort, again significantly differentiated MDD patients from controls (t = 2.394, d.f. = 23, p = 0.0125, *d* = 0.998) and BPD patients (t = 2.674, d.f. = 23, p = 0.007, *d* = 1.115) in the validation cohort, see Figure 1. Lowered transcription of *CCR6* also continued to differentiate MDD patients from controls in our validation cohort (t = −2.315, d.f. = 23, p = 0.015, *d* = 0.965), see Figure 2.

**Table 4:** A table detailing results from the one-tailed t-tests performed on our validation cohort, including t-values, degrees of freedom (d.f.), p-values, and Cohen's *d*.

Validation Cohort													
Gene	MDD v Control				MDD v BPD				BPD v Control				Replication?
	t	d.f.	p	*d*	t	d.f.	p	*d*	t	d.f.	p	*d*	
CCL24	2.394	23	**0.013**	0.998	2.674	23	0.007	1.115	-	-	-	-	Y
CCR4	−1.218	23	0.118	0.508	-	-	-	-	-	-	-	-	N
CCR6	−2.315	23	**0.015**	0.965	-	-	-	-	-	-	-	-	Y
CCR9	1.073	23	0.147	0.447	-	-	-	-	-	-	-	-	N
CXCL1	−0.455	23	0.327	0.190	-	-	-	-	-	-	-	-	N
CXCL6	−1.542	22	0.079	0.658	-	-	-	-	-	-	-	-	N
CXCL9	0.066	23	0.474	0.028	-	-	-	-	-	-	-	-	N
CXCL10	-	-	-	-	0.214	22	0.416	0.091	-	-	-	-	N
XCR1	−0.998	23	0.165	0.416	-	-	-	-	−0.879	18	0.391	0.414	N
IL8	-	-	-	-	1.331	23	0.098	0.555	−0.347	18	0.367	0.164	N
NR3C1	−0.359	23	0.362	0.150	-	-	-	-	-	-	-	-	N

Significant pairwise comparisons (p≤0.05) are highlighted in bold and indicated with a ‘Y’ under ‘Replication?’.

We additionally used one-tailed independent samples t-tests to observe whether *CCL24* and *CCR6* transcription continued to differentiate MDD patients from our other subject groups when blood was collected from a different time point (patients all in a current episode and drug-free). Again, we found that higher transcription of *CCL24* significantly differentiated MDD patients from controls in our discovery cohort (t = 7.237, d.f. = 57, p≤0.000001, *d* = 1.917) and validation cohort (t = 6.603, d.f. = 23, p≤0.000001, *d* = 2.754) when MDD blood was collected from a different time point. Similarly, we found that higher transcription of *CCL24* significantly differentiated MDD patients from BPD patients in both our discovery cohort (t = 7.247, d.f. = 57, p≤0.000001, *d* = 1.920) and validation cohort (t = 4.511, d.f. = 11.64, p≤0.001, *d* = 2.644). Additionally, lower transcription of *CCR6* continued to differentiate MDD patients from controls in both our discovery cohort (t = −1.841, d.f. = 58, p = 0.035, *d* = 0.483) and validation cohort (t = −1.799, d.f. = 23, p = 0.043, *d* = 0.750).

## Discussion

Mood disorders are heterogeneous disorders that are diagnosed when patients display a number of clinical characteristics. The absence of a specific and objective diagnostic test has led to relatively high rates of misdiagnosis for mood disorders, particularly between MDD and BPD patients [12]. Recent reports have revealed differences in cytokine gene expression between MDD patients and controls, and BPD patients and controls [30], [33]. This follows a growing body of evidence linking immuno-inflammatory processes with mood disorder pathophysiology and response to mood disorder pharmacotherapies [25], [35], [37], [39], [45], [46].

Here, we performed a small scale exploratory study which aimed to identify the presence of transcriptional differences in the inflammatory cytokine pathway between MDD, BPD and control subjects in a ‘discovery cohort’, and to assess whether these differences might act as biomarkers to differentiate between subject groups in a ‘validation cohort’. Results from our discovery cohort revealed 11 transcripts which differentiated between our subject groups (see *Table 3*). The majority of these transcripts coded for chemokines and chemokine receptors. However, two notable exceptions include interleukin-8 (*IL8*) and the glucocorticoid receptor (*NRC31*). Previous reports have found lowered levels of IL-8 protein in the blood of MDD patients relative to controls, and within the cerebrospinal fluid of suicide attempters compared to controls [47]–[49]. In the current study we found that lower transcription of *IL8* distinguished both types of mood disorder patient (MDD and BPD) from control subjects (see *Table 3*). This may suggest that a common molecular pathway impacting upon the transcription of *IL8* could be involved in mood disorder pathophysiology. We also found that MDD patients exhibited decreased transcription of *NRC31* relative to control subjects (see *Table 3*). Lowered expression of NRC31 has previously been reported both at the protein and transcriptional level amongst MDD patients, and altered expression and functionality of NRC31 has a recognized role in the pathophysiology of MDD [50]. However, neither *IL8* nor *NRC31* transcripts significantly differentiated between subject groups in our validation cohort, which suggests that although they may be involved in mood disorder pathophysiology, they were not reliable or specific enough to be utilized as biomarkers in our study.

In contrast, higher transcription of *CCL24* consistently differentiated MDD patients from control and BPD subjects, and lower transcription of *CCR6* consistently differentiated MDD patients from controls, in both our discovery and validation cohorts (see *Figure 1* and *Figure 2*). The transcription of these genes continued to differentiate MDD patients from other subject groups even when MDD blood was utilized from a different time point (see Results section), corroborating the notion that transcriptional differences in these genes likely relate to long-lasting state differences associated with MDD, as opposed to more dynamic trait differences. Furthermore, large effect sizes obtained for *CCL24* and *CCR6* (see *Tables 3* and *4*, and *Results* section) in both the discovery and validation studies support the notion that transcription of these genes could strongly differentiate MDD patients from other subject groups, and thus might indeed be useful as biomarkers.

Both *CCL24* and *CCR6* code for genes in the chemokine cytokine family. The chemokines are small chemotactic cytokines that facilitate the migration of immune cells (e.g. to a site of infection) [48]. *CCL24* codes for a chemokine which is chemotactic for resting T lymphocytes, eosinophils, and to a lesser extent neutrophils [51]–[52]. In contrast, *CCR6* codes for a G-protein coupled receptor present on immature dendritic cells, B cells and memory T cells, and binds macrophage inflammatory protein 3 alpha [53]. Chemokines have previously been implicated as potentially important cytokines in the pathophysiology of MDD and higher levels of chemokine proteins have previously been revealed amongst MDD patients relative to controls [26], [48]. However, this is the first study to identify *CCL24* and *CCR6* transcripts as potential diagnostic biomarkers.

As well as gene transcription offering a more objective method of clinical diagnosis, the fact that it is also a continuous measure gives it certain advantages over currently utilized categorical measures. For instance, continuous or dimensional diagnostic measures are believed to be more stable over time, offer a better measure of symptom severity, and be better predictors of comorbidity and chronicity [54], [55]. Subsequently, transcriptional measures, such as those reported here, could be combined with phenomenological or symptom dimension measures in future diagnostic manuals to more sensitively capture clinically useful information for MDD and BPD diagnosis.

Although results reported here are promising, there are five main limitations to this study. Firstly, this study is an exploratory study, utilizing relatively small sample sizes, and although we use both a discovery and validation cohort, patients were obtained as subsamples from the same studies, so it only offers a pseudo-independent replication. Therefore replication studies are required in a larger independent sample. Secondly, although we considered the effects of different medications on gene expression profiles, all of our patients were medicated. Based on our previous work on the MDD patient sample used here, we can, with some confidence, rule out the confounding effects of escitalopram treatment [39]. This was further supported by analyses on our MDD patients after they were medication-free for two weeks (see *Results* section). However, our BPD patient cohort were all treated with a variety of medications, and although we could rule out the confounding effects of each medication separately, we could not assess whether common actions of different medications may have confounding effects on gene transcription in our sample. Subsequently, future studies in drug-free patients are required in order to validate the transcript biomarkers identified in this study. Thirdly, although we accounted for differences in age, sex, BMI, cardiovascular problems and diabetes between our subject groups, we did not have an extensive account of comorbidities or information on smoking or alcohol drinking habits. Comorbid ailments such as chronic pain, irritable bowel syndrome and arthritis are also known to be more frequent amongst mood disorder patients and may affect cytokine expression [56], subsequently a more extensive list of comorbid disorders should be accounted for in future studies. Fourth, time of day and seasonality have previously been found to affect serum levels of cytokines, therefore this may act as a possible confounding factor. Finally, without cell count information we cannot determine the cell types in blood that may be driving our observed transcript differences between subjects.

Despite its limitations, the current study utilizes well-characterized clinical samples, stringent quality control steps, normalization protocols and statistical analyses. This study supports previous reports of differences in the expression of *IL8* and *NR3C1* amongst mood disorder patients. However, the lack of replication in our validation cohort suggests that differences in the transcription of these genes may not be reliable enough to be utilized as biomarkers. Instead, this study emphasized the potential importance of chemokines as biomarkers, and specifically it identifies the potential utility of *CCL24* and *CCR6* transcripts as novel biomarkers differentiating MDD patients from control subjects and BPD patients. Consequently, this study provides preliminary evidence that *CCL24* and *CCR6* could be used in conjunction with symptom measures to more accurately diagnose MDD from the outset and differentiate MDD patients from non-depressed subjects and BPD patients. Further replication studies are now required in a larger medication-naïve cohort to further validate these findings.

## Supporting Information

Figure S1 **A bar chart showing the results of RefFinder analyses performed on a panel of five housekeeping genes.** The gene names of the housekeeping genes are indicated on the x-axis, along with their expression stability score shown on the y-axis. Lower RefFinder stability scores represent more stable reference genes.
(DOC)

Table S1 **A table showing raw C** ***_t_*** **values generated for our genes of interest in our total subject sample.**
(XLS)

Table S2 **Relative expression (ΔC** ***_t_*** **) values for all subjects, adjusted for PPC, age, sex, current mood status and the presence of comorbid disorders (diabetes, cardiovascular problems).**
(XLS)

Table S3 **ANOVA results comparing our three subject groups.**
(DOC)

Table S4 **Games-Howell pairwise post-hoc results.**
(DOC)
