# Supplementary material for: Putative Transcriptomic Biomarkers in the Inflammatory Cytokine Pathway Differentiate Major Depressive Disorder Patients from Control Subjects and Bipolar Disorder Patients
Source: PLoS One. 2014 Mar 11;9(3):e91076. doi: 10.1371/journal.pone.0091076 (PMC3949789; doi:10.1371/journal.pone.0091076)
Supplement: Figure S1 — A bar chart showing the results of RefFinder analyses performed on a panel of five housekeeping genes. The gene names of the housekeeping genes are indicated on the x-axis, along with their expression stability score shown on the y-axis. Lower RefFinder stability scores represent more stable reference genes. (DOC) [file pone.0091076.s001.doc]

**S1: RefFinder Results**


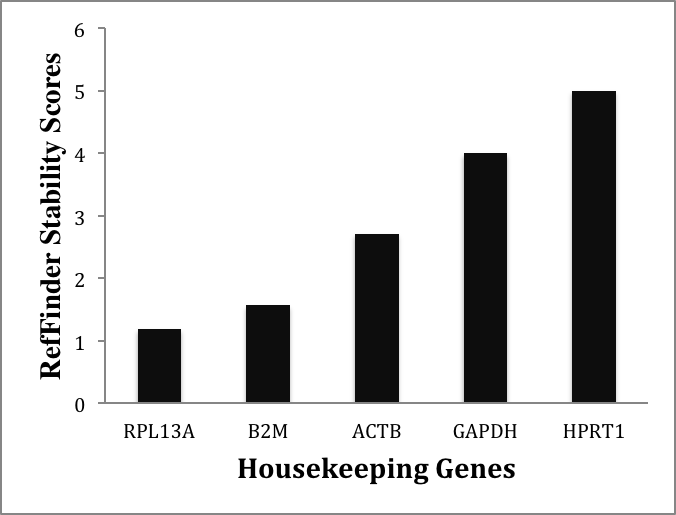


S1: A bar chart showing the results of RefFinder analyses performed on a panel of five housekeeping genes. The gene names of the housekeeping genes are indicated on the x-axis, along with their expression stability score shown on the y-axis. Lower RefFinder stability scores represent more stable reference genes.
