# Supplementary material for: Putative Transcriptomic Biomarkers in the Inflammatory Cytokine Pathway Differentiate Major Depressive Disorder Patients from Control Subjects and Bipolar Disorder Patients
Source: PLoS One. 2014 Mar 11;9(3):e91076. doi: 10.1371/journal.pone.0091076 (PMC3949789; doi:10.1371/journal.pone.0091076)
Supplement: Table S3 — ANOVA results comparing our three subject groups. (DOC) [file pone.0091076.s004.doc]

**S3: ANOVA Results**
